# Supplementary material for: Longitudinal association between handgrip strength, gait speed and risk of serious falls in a community-dwelling older population
Source: PLoS One. 2023 May 8;18(5):e0285530. doi: 10.1371/journal.pone.0285530 (PMC10166501; doi:10.1371/journal.pone.0285530)
Supplement: S1 File — (DOCX) [file pone.0285530.s001.docx]

# Longitudinal association between handgrip strength, gait speed and risk of serious falls in a community-dwelling older population

Thao Pham, John J McNeil, Anna L Barker, Suzanne G Orchard, Anne B Newman, Catherine Robb, Michael E Ernst, Sara Espinoza, Robyn L Woods, Mark R Nelson, Lawrence Beilin, Sultana Monira Hussain

**S1 Table: Baseline characteristics of the included participants overall and according to categories of gait speed.**

**S2 Table:** **Association between grip strength and risk of falls (HR, 95% CI) using European Working Group on Sarcopenia in Older People (EWGSOP) cut-off points for grip strength.**

**S3 Table: Association between grip strength and serious falls according to BMI category and sex. (HR, 95% CI)**

**S4 Table: Association between grip strength and risk of serious falls. (HR, 95% CI) excluding participants who were prefrail/frail at enrolment. (HR, 95% CI)**

**S5 Table: Association between gait speed and risk of serious falls (HR, 95% CI) excluding participants utilising walking aids. (HR, 95% CI)**

**S1 Fig. Distribution of grip strength according to sex.**

**S2 Fig. Distribution of gait speed according to sex.**

**S3 Fig. Schematic diagram of the included participants.**

**S1 Table: Baseline characteristics of the included participants overall and according to categories of gait speed.**

|  | **Overall** | **High** | **Medium** | **Low** | **p-value** |
| --- | --- | --- | --- | --- | --- |
| **N (%)** | 16616 | 3316 (20.0) | 9976 (60.0) | 3324 (20.0) |  |
| **Age in years*** | 75.3 (4.4) | 73.9 (3.3) | 75.1 (4.1) | 77.4 (5.2) | <0.001 |
| **Females, n (%)** | 9125 (54.9) | 1819 (54.9) | 5481 (54.9) | 1825 (54.9) | 0.996 |
| **Low activity, n (%)*** | 1065 (6.4) | 135 (4.1) | 544 (5.5) | 386 (11.6) | <0.001 |
| **Body mass index (kg/m^2^)*** | 28.0 (4.6) | 26.9 (3.9) | 27.9 (4.4) | 29.2 (5.4) | <0.001 |
| **Waist Circumference (cm)*** | 97.1 (12.7) | 94.3 (11.6) | 97.0 (12.4) | 100.1 (13.7) | <0.001 |
| **Smoking history, n (%)** |  |  |  |  | <0.001 |
| **Current/Former** | 7371 (44.4) | 1353 (40.8) | 4472 (44.8) | 1546 (46.5) |  |
| **Never** | 9245 (55.6) | 1963 (59.2) | 5504 (55.2) | 1778 (53.5) |  |
| **Alcohol use, n (%)** |  |  |  |  | <0.001 |
| **Current/Former** | 13925 (83.8) | 2879 (86.8) | 8386 (84.1) | 2660 (80.0) |  |
| **Never** | 2691 (16.2) | 437 (13.2) | 1590 (15.9) | 664 (20.0) |  |
| **Short Form 12 (state of health), n (%)** |  |  |  |  | <0.001 |
| **Good health** | 15910 (95.8) | 3261 (98.3) | 9644 (96.7) | 3005 (90.5) |  |
| **Fair/Poor health** | 700 (4.2) | 55 (1.7) | 328 (3.3) | 317 (9.5) |  |
| **Systolic blood pressure** | 139.8 (16.3) | 138.7 (16.1) | 139.8 (16.2) | 140.7 (17.0) | 0.001 |
| **Chronic kidney disease, n (%)†** | 12462 (75.0) | 2299 (69.3) | 7515 (75.3) | 2648 (79.7) | <0.001 |
| **Polypharmacy, n (%)‡** | 4331 (26.1) | 594 (17.9) | 2456 (24.6) | 1281 (38.5) | <0.001 |
| **On trial medication (aspirin, 100mg), n (%)** | 8280 (49.8) | 1693 (51.1) | 4914 (49.3) | 1673 (50.3) | 0.16 |
| **Exposure** | | | | | |
| **Gait speed (median and IQR), males** | 1.052 (0.304, 2.000) | 1.052 (1.003, 1.103) | 0.951 (0.893, 1.002) | 0.814 (0.339, 0.893) |  |
| **Gait speed (median and IQR), females** | 1.000 (0.323, 1.905) | 1.000 (0.952, 1.049) | 0.893 (0.830, 0.952) | 0.738 (0.323, 0.829) |  |
| **Outcome** |  |  |  |  |  |
| **Falls, n (%)*** | 1533 (9.2) | 231 (7.0) | 846 (8.5) | 457 (13.8) | <0.001 |

^⁙^Gait speed (m/s) categories: low (Q 1, lowest 20%), medium (Q 2-4, middle 60%), high (Q 5, highest 20%)

†Chronic kidney disease baseline: EGFR > an estimated glomerular filtration rate of less than 60 ml per minute per 1.73 m2 or a ratio of albumin (in milligrams per litre) to creatinine (in millimoles per litre) in urine of 3 or more

‡Polypharmacy: the use of >5 medications

**S2 Table Association between grip strength and risk of falls (HR, 95% CI) using European Working Group on Sarcopenia in Older People (EWGSOP) cut-off points for grip strength**

|  | **Model 1** | **Model 2** | **Model 3** | **Model 4** |
| --- | --- | --- | --- | --- |
| **Stratified by sex** | | | | |
| **Males**** **(n=7430)** | | | | |
| **For all (each SD decrease)** | 1.32 (1.18, 1.48) | 1.30 (1.17, 1.46) | 1.28 (1.14, 1.44) | 1.28 (1.14, 1.44) |
| **Grip strength (kgf) as categorical variable** | | | | |
| **Normal** | Ref | Ref | Ref | Ref |
| **Abnormal** | 1.45 (1.17, 1.79) | 1.43 (1.15, 1.77) | 1.35 (1.08, 1.69) | 1.35 (1.08, 1.69) |
| **Females**(n= 9015)** | | | | |
| **For all (each SD decrease)** | 1.32 (1.18, 1.48) | 1.30 (1.16, 1.46) | 1.27 (1.43, 1.13,) | 1.27 (1.12, 1.43) |
| **Grip strength (kgf) as categorical variable** | | | | |
| **Normal** | Ref | Ref | Ref | Ref |
| **Abnormal** | 1.28 (1.11, 1.48) | 1.25 (1.08, 1.45) | 1.21 (1.04, 1.40) | 1.20 (1.04, 1.40) |

Kgf = kilogram force; SD = standard deviation; Ref = reference.

Data presented as hazard ratio [HR, 95% confidence interval (CI)]. Model 1: age and gender. Model 2: age, gender, physical activity, BMI and self-reported health status. Model 3: age, gender, physical activity, BMI, SF12 State of health chronic kidney disease, and polypharmacy. Model 4: age, gender, physical activity, BMI, SF12 State of health, chronic kidney disease, polypharmacy and aspirin (100mg).

*Grip strength (kgf) categories: females; normal ≥16 kgf, abnormal <16 kfg. males; normal ≥27 kgf; abnormal <27 kgf

**Not adjusted for gender

**S3 Table: Association between grip strength and serious falls according to BMI category and sex. (HR, 95% CI)**

|  | **Model 1** | **Model 2** | **Model 3** | **Model 4** |
| --- | --- | --- | --- | --- |
| **Male^⁂^** | | | | |
| **Normal weight* (n=1622)** | | | | |
| **Grip strength (each SD decrease)** | 1.13 (0.89, 1.44) | 1.14 (0.89, 1.45) | 1.11 (0.87, 1.43) | 1.11 (0.87, 1.42) |
| **Grip strength as categorical variable^⁜^** | | | | |
| **High ( 5)** | Ref | Ref | Ref | Ref |
| **Medium (Q 2-4)** | 1.85 (0.92. 3.72) | 1.85 (0.92, 3.72) | 2.00 (0.96, 4.19) | 1.97 (0.94, 4.12) |
| **Low (Q 1)** | 2.31 (1.09, 4.89) | 2.30 (1.08, 4.89) | 2.37 (1.07, 5.24) | 2.34 (1.06, 5.17) |
| **Overweight** (n=3875)** | | | | |
| **Grip strength (each SD decrease)** | 1.38 (1.17, 1.62) | 1.36 (1.15, 1.61) | 1.35 (1.10, 1.65) | 1.33 (1.11, 1.58) |
| **Grip strength as categorical variable^⁜^** | | | | |
| **High (Q 5)** | Ref | Ref | Ref | Ref |
| **Medium (Q 2-4)** | 1.63 (1.05, 2.52) | 1.62 (1.04, 2.50) | 1.81 (1.23, 2.92) | 1.80 (1.12, 2.90) |
| **Low (Q 1)** | 2.05 (1.26, 3.32) | 1.99 (1.22, 3.23) | 2.05 (1.21, 3.47) | 2.05 (1.21, 3.47) |
| **Obese*** (n=1907)** | | | | |
| **Grip strength (each SD decrease)** | 1.38 (1.13, 1.68) | 1.34 (1.10, 1.63) | 1.35 (1.10, 1.65) | 1.35 (1.10, 1.65) |
| **Grip strength as categorical variable^⁜^** | | | | |
| **High (Q5)** | Ref | Ref | Ref | Ref |
| **Medium (Q 2-4)** | 1.92 (1.08, 3.39) | 1.89 (1.07, 3.35) | 2.05 (1.11, 2.78) | 2.05 (1.11, 3.78) |
| **Low (Q1)** | 2.56 (1.38, 4.76) | 2.45 (1.31, 4.56) | 2.58 (1.33, 5.01) | 2.59 (1.33, 5.03) |
| **Female^⁂^** | | | | |
| **Normal weight* (n=2686)** | | | | |
| **Grip strength (each SD decrease)** | 1.22 (0.99, 1.51) | 1.22 (0.99, 1.50) | 1.16 (0.93, 1.44) | 1.15 (0.93, 1.43) |
| **Grip strength as categorical variable^⁜^** | | | | |
| **High (Q5)** | Ref | Ref | Ref | Ref |
| **Medium (Q 2-4)** | 1.61 (1.09, 2.37) | 1.62 (1.10, 2.38) | 1.61 (1.08, 2.40) | 1.60 (1.07, 2.39) |
| **Low (Q1)** | 1.63 (1.06, 2.49) | 1.62 (1.06, 2.48) | 1.55 (1.00, 2.41) | 1.54 (0.99, 2.40) |
| **Overweight** (n=3484)** | | | | |
| **Grip strength (each SD decrease)** | 1.29 (1.07, 1.55) | 1.25 (1.04, 1.50) | 1.21 (1.00, 1.47) | 1.22 (1.00, 1.48) |
| **Grip strength as categorical variable^⁜^** | | | | |
| **High (Q5)** | Ref | Ref | Ref | Ref |
| **Medium (Q 2-4)** | 1.10 (0.81, 1.49) | 1.07 (0.79, 1.46) | 1.04 (0.75, 1.43) | 1.09 (0.76, 1.43) |
| **Low (Q 1)** | 1.46 (1.03, 2.05) | 1.39 (0.98, 1.95) | 1.31 (0.92, 1.88) | 1.32 (0.92, 1.89) |
| **Obese** (n=2798)** | | | | |
| **Grip strength (each SD decrease)** | 1.47 (1.20, 1.80) | 1.45 (1.18. 1.78) | 1.47 (1.19, 1.82) | 1.47 (1.19, 1.82) |
| **Grip strength (kilogram force) as categorical variable^⁜^** | | | | |
| **High (Q5)** | Ref | Ref | Ref | Ref |
| **Medium (Q2-4)** | 1.37 (0.95, 1.99) | 1.37 (0.95, 2.79) | 1.45 (0.99, 2.14) | 1.44 (0.98, 2.13) |
| **Low (Q 1)** | 1.88 (1.25, 2.83) | 1.85 (1.23, 2.79) | 1.94 (1.26, 2.98) | 1.94 (1.26, 2.98) |

SD = standard deviation; Ref = reference. Data presented as hazard ratio [HR, 95% confidence interval (CI)]. Model 1 was adjusted for age and gender. Model 2 was additionally adjusted for age, gender, physical activity, body mass index (kg/m^2^) and Short Form 12 (state of health). Model 3 was additional adjusted for age, gender, physical activity, body mass index (kg/m^2^), Short Form 12 (state of health), chronic kidney disease, and polypharmacy. Model 4 was additional adjusted for age, gender, physical activity, body mass index (kg/m^2^), Short Form 12 (state of health), chronic kidney disease, polypharmacy and aspirin (100mg).

^⁜^Grip strength (kilogram force) categories: low (Q1, lowest 20%), medium (Q2-4, middle 60%), high (Q5, highest 20%)

**^⁂^**Not adjusted for gender.

*Normal weight: BMI <25

**Overweight: 25.0 to <30

***Obesity: BMI 30.0 or higher.

**S4 Table: Association between grip strength and risk of serious falls (HR, 95% CI) excluding participants who were prefrail/frail at enrolment. (HR, 95% CI)**

|  | **Model 1** | **Model 2** | **Model 3** | **Model 4** |
| --- | --- | --- | --- | --- |
| **All population (n=10 145)** | | | | |
| **For all population (each SD decrease)** | 1.45 (1.24, 1.70) | 1.44 (1.22, 1.69) | 1.44 (1.22, 1.70) | 1.44 (1.22, 1.70) |
| **Grip strength as categorical variable^⁜^** | | | | |
| **High (Q 5)** | Ref | Ref | Ref | Ref |
| **Medium (Q 2-4)** | 1.41 (1.16, 1.72) | 1.40 (1.15, 1.71) | 1.43 (1.16, 1.76) | 1.43 (1.16, 1.76) |
| **Low (Q 1)** | 1.97 (1.29, 3.00) | 1.87 (1.20, 2.89) | 1.89 (1.20, 2.96) | 1.89 (1.20, 2.96) |
| **Male (n=4668) ^⁂^** | | | | |
| **For all population (each SD decrease)** | 1.45 (1.16, 1.81) | 1.44 (1.15, 1.81) | 1.45 (1.15, 1.84) | 1.45 (1.15, 1.83) |
| **Grip strength as categorical variable^⁜^** | | | | |
| **High (Q 5)** | Ref | Ref | Ref | Ref |
| **Medium (Q 2-4)** | 1.63 (1.14, 2.33) | 1.61 (1.13, 2.31) | 1.68 (1.15, 2.44) | 1.68 (1.15, 2.44) |
| **Low (Q 1)** | 3.32 (1.67, 6.62) | 3.30 (1.63, 6.67) | 3.53 (1.72, 7.23) | 3.54 (1.72, 7.25) |
| **Female (n=5477) ^⁂^** | | | | |
| **For all population (each SD decrease)** | 1.46 (1.17, 1.84) | 1.44 (1.14, 1.81) | 1.44 (1.14, 1.93) | 1.44 (1.14, 1.83) |
| **Grip strength as categorical variable^⁜^** | | | | |
| **High (Q 5)** | Ref | Ref | Ref | Ref |
| **Medium (Q 2-4)** | 1.32 (1.05, 1.67) | 1.32 (1.04, 1.67) | 1.33 (1.04, 1.71) | 1.33 (1.04, 1.71) |
| **Low (Q 1)** | 1.53 (0.89, 2.62) | 1.39 (0.78, 2.46) | 1.38 (0.76, 2.49) | 1.38 (0.76, 2.49) |

Note. SD = standard deviation; Ref = reference. Data presented as hazard ratio [HR, 95% confidence interval (CI)]. Model 1 was adjusted for age and gender. Model 2 was additionally adjusted for age, gender, physical activity, body mass index (kg/m^2^) and Short Form 12 (state of health). Model 3 was additional adjusted for age, gender, physical activity, body mass index (kg/m^2^), Short Form 12 (state of health), chronic kidney disease, and polypharmacy. Model 4 was additional adjusted for age, gender, physical activity, body mass index (kg/m^2^), Short Form 12 (state of health), chronic kidney disease, polypharmacy and aspirin (100mg).

^⁜^Grip strength (kilogram force) categories: low (Q 1, lowest 20%), medium (Q 2-4, middle 60%), high (Q 5, highest 20%)

**^⁂^**Not adjusted for gender

**S5 Table: Association between gait speed and risk of serious falls (HR, 95% CI) excluding participants utilising walking aids. (HR, 95% CI)**

|  | **Model 1** | **Model 2** | **Model 3** | **Model 4** |
| --- | --- | --- | --- | --- |
| **All population (n=10 145)** | | | | |
| **For all population (each SD lower)** | 0.998 (0.997, 0.999) | 0.998 (0.997, 0.999) | 0.998 (0.997, 0.999) | 0.998 (0.997, 0.999) |
| **Gait speed (m/s) as categorical variable^⁙^** | | | | |
| **High (Q 5)** | Ref | Ref | Ref | Ref |
| **Medium (Q 2-4)** | 1.16 (1.00, 1.34) | 1.15 (0.99, 1.34) | 1.17 (1.00, 1.36) | 1.17 (1.00, 1.36) |
| **Low (Q 1)** | 1.52 (1.29, 1.80) | 1.47 (1.23, 1.74) | 1.44 (1.21, 1.73) | 1.44 (1.21, 1.73) |
| **Male^⁂^ (n=4668)** | | | | |
| **For all population (each SD lower)** | 0.996 (0.995, 0.998) | 0.997 (0.995, 0.999) | 0.997 (0.996, 0.999) | 0.997 (0.996, 0.999) |
| **Gait speed (m/s) as categorical variable^⁙^** | | | | |
| **High (Q 5)** | Ref | Ref | Ref | Ref |
| **Medium (Q 2-4)** | 1.28 (0.98, 1.66) | 1.24 (0.95, 1.61) | 1.26 (0.96, 1.65) | 1.26 (0.96, 1.65) |
| **Low (Q 1)** | 1.68 (1.25, 2.26) | 1.54 (1.14, 2.08) | 1.52 (1.11, 2.07) | 1.52 (1.11, 2.07) |
| **Female^⁂^ (n=5477)** | | | | |
| **For all population (each SD lower)** | 0.998 (0.997, 0.999) | 0.998 (0.997, 0.999) | 0.998 (0.997, 0.999) | 0.998 (0.997, 0.999) |
| **Gait speed (m/s) as categorical variable^⁙^** | | | | |
| **High (Q 5)** | Ref | Ref | Ref | Ref |
| **Medium (Q 2-4)** | 1.10 (0.92, 1.31) | 1.11 (0.93, 1.33) | 1.13 (0.94, 1.36) | 1.13 (0.94, 1.36) |
| **Low (Q 1)** | 1.45 (1.18, 1.78) | 1.44 (1.16, 1.78) | 1.42 (1.13, 1.77) | 1.42 (1.13, 1.77) |

m/s = metre per second; SD = standard deviation; Ref = reference. Data presented as hazard ratio [HR, 95% confidence interval (CI)]. Model 1 was adjusted for age and gender. Model 2 was additionally adjusted for age, gender, physical activity, body mass index (kg/m^2^) and Short Form 12 (state of health). Model 3 was additionally adjusted for age, gender, physical activity, body mass index (kg/m^2^), Short Form 12 (state of health), chronic kidney disease, and polypharmacy. Model 4 was additionally adjusted for age, gender, physical activity, body mass index (kg/m^2^), Short Form 12 (state of health), chronic kidney disease, polypharmacy and aspirin (100mg).

^⁙^Gait speed (m/s) categories: low (Q 1, lowest 20%), medium (Q 2-4, middle 60%), high (Q 5, highest 20%)

**^⁂^**Not adjusted for gender

**S1 Fig. Distribution of grip strength according to sex.**


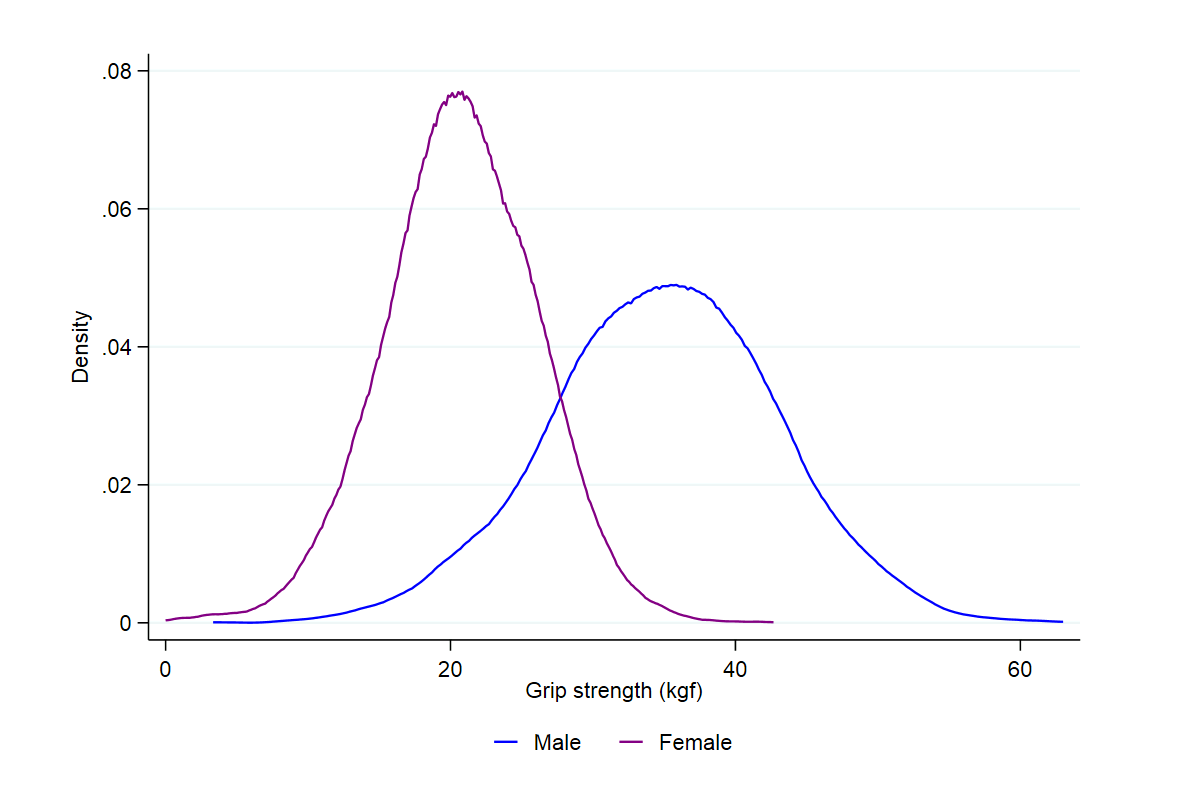


**S2 Fig. Distribution of gait speed according to sex.**


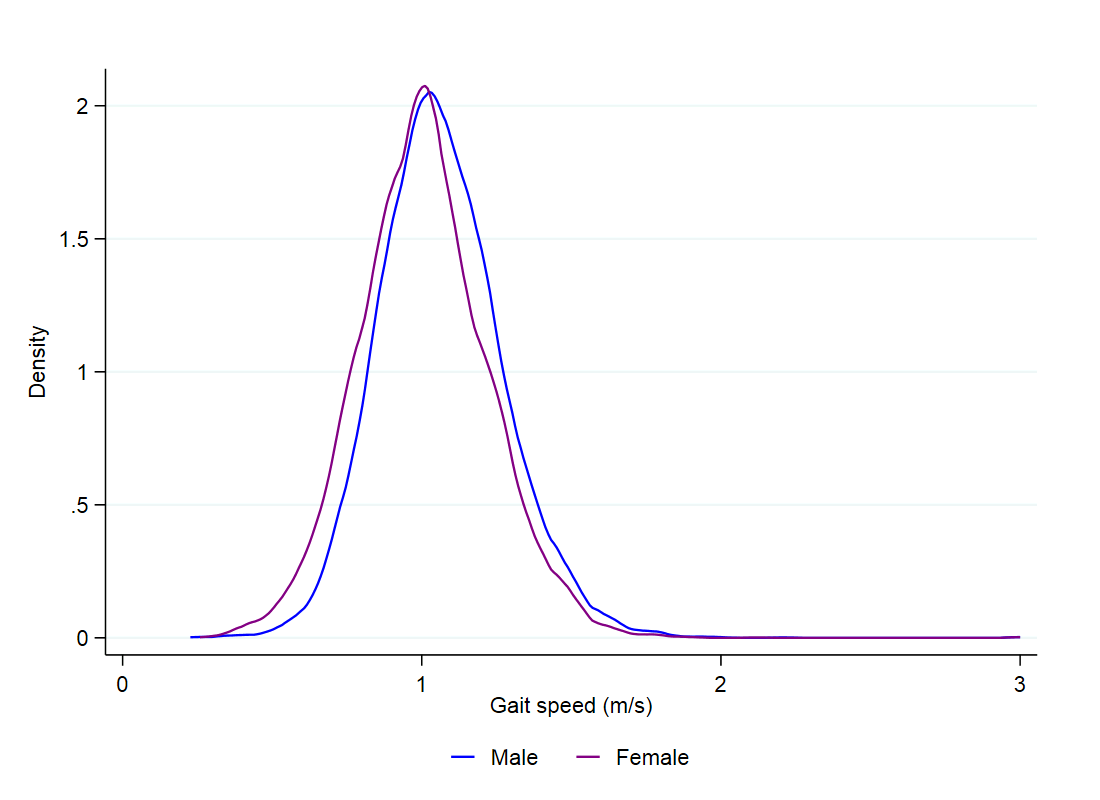


**S3 Fig. Schematic diagram of the included participants.**

ASPREE sample

N=19 114

US participants not included in the sub-study

N=2411

Randomized in the ASPREE-Fracture sub-study

16 703 including 1539 Falls

Grip strength not measured

N=258

Gait speed not measured

N=87

16 445 with grip strength included in the current analyses with 1512 falls

16 616 with gait speed included in the current analyses with 1533 falls
